# Neuropilin-1 in Transplantation Tolerance

**DOI:** 10.3389/fimmu.2013.00405

**Published:** 2013-11-25

**Authors:** Mauricio Campos-Mora, Rodrigo A. Morales, Tania Gajardo, Diego Catalán, Karina Pino-Lagos

**Affiliations:** ^1^Immune Regulation and Tolerance Research Group, Programa Disciplinario de Inmunología, Facultad de Medicina, Instituto de Ciencias Biomédicas, Universidad de Chile, Santiago, Chile

**Keywords:** Foxp3, neuropilin-1, tolerance, transplantation, Tregs

## Abstract

In the immune system, Neuropilin-1 (Nrp1) is a molecule that plays an important role in establishing the immunological synapse between dendritic cells (DCs) and T cells. Recently, Nrp1 has been identified as a marker that seems to distinguish natural T regulatory (nTreg) cells, generated in the thymus, from inducible T regulatory (iTreg) cells raised in the periphery. Given the crucial role of both nTreg and iTreg cells in the generation and maintenance of immune tolerance, the ability to phenotypically identify each of these cell populations *in vivo* is needed to elucidate their biological properties. In turn, these properties have the potential to be developed for therapeutic use to promote immune tolerance. Here we describe the nature and functions of Nrp1, including its potential use as a therapeutic target in transplantation tolerance.

## Introduction

**Figure 1:**
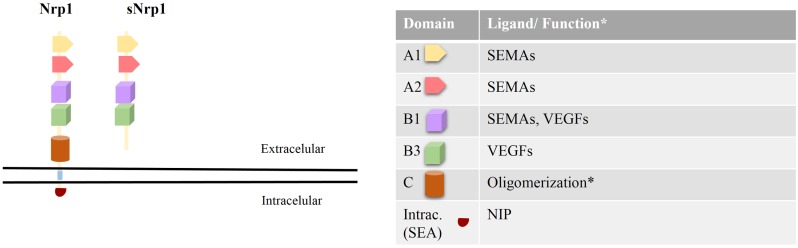
**Neuropilin-1 structure, ligands, and function**. Left: Nrp1 is comprised of five extracellular domains (a1/a2, b1/b2, and c), one transmembrane domain, and a short cytoplasmic domain. The a1/a2 and b1/b2 domains are involved in binding to several ligands, while the c- and transmembrane domains are involved in receptor oligomerization. The intracellular domain of Nrp1 interacts with PDZ domain proteins (such as synectin) via its C-terminal recognition sequence S-E-A. In addition, a natural-occurring soluble form of Nrp1 (sNrp1), lacking the c- and transmembrane domains, is shown. Right: the function and ligand binding properties of each Nrp1 domain is depicted.

Neuropilin-1 (Nrp1) is a 120–130 kDa type-I transmembrane glycoprotein with a multi-domain extracellular region, a single transmembrane helix, and a cytoplasmic domain. The extracellular region consists of two CUB domains (denoted a1/a2), two coagulation factor V/VIII homology domains (denoted b1/b2), and a MAM domain (denoted c) (1). Previous reports suggest that the a1/a2 and b1/b2 domains are involved in the binding of Nrp1 to its ligands (2, 3), whereas the c- and transmembrane domains participate in receptor dimerization (4). The intracellular domain interacts with PDZ domain proteins (5), yet its function is not defined (Figure 1).

Neuropilin-1 was first described to be involved in the development of neurons (6–11), where it plays an important role as co-receptor of various molecules, as described below (12–19). More recent, immunologists have been interested in Nrp1 due to its role in the formation of the immune synapse between dendritic cells (DCs) and T cells, leading to the activation of T cells (20–23).

In addition to its expression in different cell types, an important feature of Nrp1 is its ability to bind many different ligands; these ligands control a variety of biological processes through binding to Nrp1 (13–19). In this review, we discuss the identification of Nrp1 and its biology, and then focus on the relevance of Nrp1 in the immune system and its potential clinical use in transplant tolerance.

### Neuropilin-1 biology

Initially known as A5, Nrp1 was identified in the late 80s as an antigen preferentially expressed on superficial layers of the optic tectum of *Xenopus laevis* through screening candidate molecules implicated in retinotectal projection development (6–8). The composition of these layers predominantly consist of synapses, glial processes, dendrites and axonal ends of retinal neurons, or “neuropiles,” and therefore termed Neuropilin-1 (8).

During the 90s, research on Nrp1 was limited to the field of developmental biology. In mice, Nrp1 is expressed in olfactory, hippocampal, retinal, and sensory peripheral neurons; its expression varies according to the development stage and establishment of neuronal circuits (9, 10). Signaling through Nrp1 expressed on neurons promotes neurite outgrowth *in vitro*, which can be inhibited using anti-Nrp1 antibodies (24). In addition, systemic overexpression of Nrp1 under control of the β-actin promoter leads to embryonic death due to several morphological abnormalities, such as anomalous sprouting, defasciculation of nervous fibers, and cardiovascular abnormalities like excessive formation of capillaries, blood vessels, and heart malformation (11). Together, these observations suggest an important role for Nrp1 in embryonic vessel formation and neuronal interactions.

Several investigators subsequently reported that Nrp1 is a class III semaphorin (Sema3) receptor (discussed later), confirming an important role for Nrp1 in axonal guidance (25–27). These findings are consistent with Nrp1 being identified as a novel vascular and endothelial growth factor (VEGF) receptor (12), and a Nrp1 deficiency in mice resulting in embryonic lethality at 10–12.5 days due to severe anomalies in the vascular system, including impaired neural vascularization, absence and/or transposition of important vessels and disorganization of the extra embryonic vasculature (28). Taken together, these results demonstrated that Nrp1 is essential for normal embryological development of the nervous and cardiovascular systems.

Neuropilin-1 has a homologous protein called Neuropilin-2 (Nrp2), which shares 44% peptide sequence homology (26, 29). Both proteins have similar molecular weight, general domain structure, and share certain ligand specificities (30, 31). Furthermore, they can form Nrp1/Nrp2 heterodimers (32, 33). Nrp1 and Nrp2 are present only in vertebrates, despite the evidence indicating the existence of Nrp ligands in some invertebrates (30, 34). In addition to membrane-bound Nrp1 and Nrp2, several secreted forms of these proteins, which lack the transmembrane and cytoplasmic domains, have been identified and sought to act as natural inhibitors (35–39).

A notable feature of Nrp1 and Nrp2 is their ability to bind with relatively high affinity to several families of molecules, which involves them in a variety of physiological processes in a manner that is not yet fully understood. These molecules include Sema3 and heparin-binding members of the VEGF family that bind to Nrp1. VEGF family members are potent angiogenic molecules with chemotactic, survival, and proliferating effects in endothelial cells (40). Nrp1 is a functional receptor for specific members of the family of these angiogenic factors acting as a co-receptor together with VEGF-receptors (12). In addition, Nrp1 binds to hepatocyte growth factor (HGF and its receptor c-Met) (13, 14), some members of the fibroblast growing factor (FGF) family (13), platelet derived growth factor (PDGF and its receptors) (15, 16), and Galectin-1 (Gal-1) (17). Finally, Nrp1 interacts with integrins (18, 19) and even binds with itself (13), implicating a role in immune cells (discussed later).

Neuropilin-1 is expressed in epithelial cells from different tissues (e.g., gastrointestinal tract, pancreas, thymus), neurons, melanocytes, and keratinocytes (31, 41). In many cases, Nrp1 participates in the generation of various types of organs, which can be attributed largely to its role as a VEGF receptor. For example, Nrp1 is involved in pancreatic islet neogenesis and its expression is restricted to islet cells (42). Moreover, Nrp1 expression levels increase during lung organogenesis and its expression remains in normal alveolar epithelium (43). Nrp1 also participates in glomerulogenesis and wound repair in renal glomerular and keratinocytes, respectively (44, 45).

Neuropilin-1 is expressed by a variety of human tumor cell types (46), such as breast cancer cells, melanoma, astrocytoma, and prostate carcinoma, among others (12, 47–49). Although in certain clinical studies it was found a correlation between Nrp1 expression and increased hypervascularity, malignancy, and/or aggressiveness (46), sNRP1 (a soluble form of Nrp1) had a antitumoral effect (35).

### Semaphorins and their relationship with Nrp1 in immune cells

**Figure 2:**
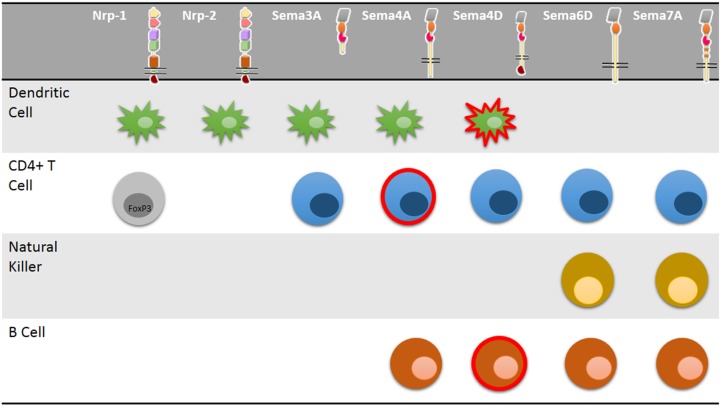
**Neuropilin and semaphorin expression in immune cells**. Multiple immune cells express neuropilins and semaphorins and signaling through these molecules control distinct cellular properties including cell migration, activation, or modulation of immune cell response and CD4+ T-cell polarization. Some neuropilins and semaphorins are uniquely expressed in specific CD4+ T-cell subsets such as activated cells (red lined cells) or FoxP3+ regulatory T cells (gray cell).

Semaphorins are a family of either membrane-associated or soluble proteins that share a common structural domain known as a *Sema* domain (50). This family is composed of ∼30 different proteins that are involved in axonal guidance, as chemorepellents of neurite growth during central nervous system (CNS) development, proliferation, and cytoskeleton organization (51), organogenesis, vascularization, angiogenesis, and cancer (50). Furthermore, some semaphorins are present in different immune cells (Figure 2), including lymphocytes, Natural Killer (NK) cells, monocytes, DCs (51). These semaphorins belong to class III, IV, VI, and VII, and their roles in immune regulation are discussed below.

#### Class VII semaphorins

Sema7A is a membrane-attached glycoprotein (also known as CD108), which is expressed on activated lymphocytes and thymocytes. Its receptor is VESPR/CD232/plexin-C1, present in monocytes and macrophages (52). Sema7A signaling induces the production of pro-inflammatory cytokines such as TNF-α, IL-6, and IL-8, and the chemotaxis of monocytes (53). Studies using genetically manipulated mice suggested an important role for Sema7A in some immune diseases. For example, *Sema7a*-knockout mice are resistant to inflammation and it has been described that Sema7A is an important molecule in the pathogenesis of lung fibrosis, exacerbating fibrosis via binding to β1-integrin (54). Furthermore, signals through Sema7A on monocytes promotes the production of granulocyte-macrophage colony stimulating factor (GM-CSF), inducing morphological changes transforming them to a DCs-like morphology (55).

#### Class VI semaphorins

Sema6D is expressed on T cells, B cells, and NK cells. Its receptor is plexin-A1, known for being expressed specifically on DCs. Studies have demonstrated that recombinant Sema6D recognizes and binds to plexin-A1 on activated DCs, increasing IL-12 production. Additionally, Plexin-A1-knockout mice exhibit impaired T-cell responses, being resistant to experimental autoimmune encephalomyelitis (EAE). Specifically, it has been shown that Sema6D expression increases belatedly during CD4+ T-cell activation, and its blockade causes an impaired late-phase T-cell proliferation and the inhibition of CD127 expression (56). Also, it has been found that Sema6D is present in gastric carcinoma tissue where it would be playing an important role inducing angiogenesis (57).

#### Class IV semaphorins

Sema4D, also known as CD100, was the first semaphorin to be associated with an immune function (58). This molecule is expressed constitutively in T cells and at low levels in resting B cells and DCs, but is upregulated in these cells upon activation (59, 60). During this event Sema4D is proteolytically cleaved and is released as a soluble protein (55), which has been implicated in physiological and pathological immune responses (54). Soluble Sema4D has been detected in immunized mice (50). *Sema4d*-knockout mice have impaired humoral responses against T-cell-dependent antigens (59), and fail to develop EAE caused by an impaired priming of T cells by DCs, which is essential in the induction of antigen-specific T-cell responses (60).

Two receptors have been described for Sema4D: CD72 for lymphoid cells and Plexin-B1 for non-lymphoid cells (55). The cytoplasmic domain of CD72 has an immunoreceptor tyrosine-based inhibition motif (ITIM) that binds to SH2-containing tyrosine phosphatase 1 (SHP1) and acts as a negative regulator of B cell responses (61, 62). When Sema4D interacts with CD72, induces tyrosine dephosphorylation of the ITIM motif, turning off the negative signal by disrupting CD72-SHP1 interaction, and promoting positive stimulation of B cells (59, 63). It has been suggested that Sema4D could be the ligand for plexin-B1, reversing its roles and implying different functions for this semaphorin (50).

Sema4A is expressed in DCs, B cells, and activated T cells (64), especially in Th1-polarized cells (65), suggesting that Sema4A helps drive Th1 polarization. Sema4A is also expressed in Th17 cells (66). In the EAE model, mice treated with an anti-Sema4A blocking antibody were protected against the disease, as demonstrated by decreased numbers of infiltrating mononuclear cells into the CNS and diminished reactivity of myelin oligodendrocyte glycoprotein (MOG)-specific T cells (64). Analysis of *Sema4a*-deficient mice demonstrated that these animals have an impaired Th1-type response, and DCs from these mice were poor stimulators of allogeneic T cells, indicating a non-redundant role of Sem4A in DCs and T cells (65). The receptor of Sema4A in immune cells is Tim-2 (64), which is expressed by Th2-polarized cells, and its been suggested that the expression of Sema4A by Th1-polarized cells negatively regulates Th2 cell response via interaction with Tim-2 (54). Recently, it has been shown that Sema4A expressed in plasmacytoid dendritic cells (pDCs) can modulate anti-tumor responses by potentiating regulatory T-cell function and stability in the tumor microenvironment (67).

Sema4B is expressed in T cells and B cells, and promotes Th2 cell skewing (68). Supporting this observation, Sema4b^−/−^ mice have increased levels of serum IgE and an enhanced basophil-mediated IgE production. Although the receptor for Sema4B remains unidentified, it has been suggested that the regulatory properties of Sema4B in basophils would be mediated through ITIM-containing molecules (54).

#### Class III semaphorins

Sema3E is involved in thymocyte development (69). It is expressed on medullar thymic epithelial cells (mTECs), and CD4+CD8+ double positive (DP) thymocytes express high levels of Plexin-D1, the receptor for Sema3E. *Plxnd1*- and *Sema3e-*knockout mice have abnormal thymic development, with no clear cortical-medullary demarcation (69). In these mutant mice, CD69+ DP thymocytes localize in the cortical area in contrast to wild type mice where CD69+ DP thymocytes localize in the medullary zone of the thymus (69).

#### Semaphorin 3A

Sema3A acts as a negative regulator of the immune system (54), and is expressed in activated DCs and T cells, attenuating T-cell proliferation directly (70). Sema3A is also expressed in several tumor cells, inhibiting T-cell proliferation by activation of Ras/MAPK signaling pathway (71). The receptor of Sema3A is a complex formed by Nrp1 and plexin-A (72), and mutants for the receptors of Sema3A exhibit an enhanced T-cell response, both *in vitro* and *in vivo* (73). Furthermore, the *in vivo* administration of plasmid DNA encoding Sema3A reduces the severity of mice suffering from collagen-induced arthritis (CIA) (74). Given these findings, Sema3A is a potential target for the treatment of autoimmune diseases (75, 76).

Sema3A is also involved in thymocyte development and controlling the migration of immune cells (77). Acting as a chemorepellent, signaling through Sema3A impairs the migration of T cells and monocytes *in vitro* (78, 79). *In vivo*, Plexin-A1 deficient and Nrp1 defective mice have impaired T-cell responses due to the incapacity of DCs, that normally express both molecules, to transmigrate across lymphatic endothelial cells and to reach draining lymph nodes, where DCs encounter T cells to present the antigen (80). In the same work it is showed that DCs suffer morphological changes (cytoskeleton reorganization) during transmigration, which are Sema3A-dependent. These interactions would regulate DCs contractility and adhesion capabilities when passing through vessel walls (54).

In contrast to the immunosuppressive role of Sema3A, Wen et al. reported that macrophages and DCs from plexin-A4 knock out mice have defective cytokine production upon stimulation with TLR agonists. In the same work, administration of exogenous Sema3A exacerbates cytokine production in wild type cells, indicating that Sema3A is an enhancer of the innate immune response (81). Additional studies are needed to clarify the function of Sema3A in immune responses.

### Nrp1 in T-cell-mediated immunity

#### DCs and CD4+ T cells

In 2002, Tordjman et al. described for the first time that Nrp1 is expressed on human DCs. Analysis of Nrp1 expression during *in vitro* differentiation from monocyte to DCs demonstrated that Nrp1 is not expressed in monocytes but only after differentiation into DCs (20). Nrp1 expression correlated with DC-SIGN. Consistent with these findings, Nrp1 is expressed in human DCs of lymph nodes from dermatopathic lymphadenopathy patients. Interestingly, Nrp1-expressing DCs were concentrated in the T-cell-rich areas, suggesting that Nrp1 pathway can influence DC-T-cell interaction and supporting previous findings in which Nrp1 is involved in the formation of the immunologic synapse. Nrp1 is also expressed on peripheral blood T cells. Together, these observations suggest that Nrp1 plays a role in promoting cognate interactions between DC and T cells perhaps through the formation of the immunological synapse necessary to initiate the immune response. Evidence to support this hypothesis is provided from *in vitro* studies using allogeneic DC-T-cell co-cultures that demonstrated Nrp1 expression to be co-localized with CD3 expression on T cells and required for T-cell proliferation; Nrp1 blocking antibodies diminished the ∼50–60% T-cell proliferation when antibodies were directed to Nrp1 on DC or T-cell (20).

Corbel et al. characterized Nrp1 expression on murine thymocytes and other cell populations (21). First, they analyzed Nrp1 expression on both immature and mature bone marrow-derived DCs (BM-DCs) and found that ∼5% of immature BM-DCs express Nrp1 in the cell surface. This frequency increases to ∼45% of LPS-matured BM-DCs. In the thymus, ∼40–50% of leukocytes express Nrp1 (mainly by DN, DP, and CD4+CD25+ T cells) (21). In a very interesting report, it is proposed that Nrp1 expressed on human DCs can be transferred onto T cells via trogocytosis since Nrp1 expression on CD4+ T cells occurs as early as 15 min post co-culture with DCs. This was confirmed using an inhibitor for protein synthesis (in which case Nrp1 detection on T cells still occurred) and performing co-cultures with B cells (as antigen presenting cells) transduced with a vector containing a reporter (GFP) Nrp1. In these experiments, CD4+ T cells cultured with Nrp1^GFP^-B cells express Nrp1^GFP^ in the membrane, and the level of expression is directly correlated with the amount of expression on B cells. Cell-contact and membrane transfer were confirmed using transwell experiments and membrane dye assays, respectively. Their results propose an interesting mechanism by which CD4+ T cells may be acquiring Nrp1 from DCs to modulate the immune response (by binding to Nrp1 ligands or interacting with other cells), but since there was not activation of CD4+ T-cell signaling pathways upon Nrp1 binding, the mechanism and relevant *in vivo* impact of this phenomenon are still unknown (21).

#### Regulatory T cells

Two years after Tordjman’s findings, Bruder et al. studied the expression of Nrp1 on murine CD4+ T cells (22). In this work, they isolated CD4+CD25+ regulatory T cells (Tregs) and CD4+CD25− T cells (activated or not *in vitro*) and performed gene array analysis. In these experiments, Tregs could be distinguished from the CD4+CD25− T-cell counterparts by a defined group of differentially expressed genes, which contained Foxp3, KLRG1, and Nrp1. Nrp1 expression on Tregs at the plasma membrane was confirmed using antibody surface staining. Polyclonal and antigen-specific activation of Tregs *in vitro* demonstrated that the level of Nrp1 remains unchanged on Tregs, while Nrp1 expression on activated CD4+ T cells is almost lost (22). Bruder et al. also demonstrated that Nrp1 expression is directly related with both, Foxp3 expression and suppressive properties on Tregs.

Sarris et al. analyzed the contribution of Nrp1 expression on Tregs and its role in the formation of immune synapse with DCs, antigen recognition, and proliferation (23). In this report, the authors used time-lapsed video microscopy to quantify the duration of Tregs and DCs interaction. Results from these studies indicated that naïve CD4+ T cells and Tregs establish long-lasting interactions (>400 s) with DCs in a MHC-II-dependent manner, but the frequency of cells interacting with DCs was twofold higher with Tregs (∼44%) compared to naïve CD4+ T cells (∼23%). Most importantly, the long-lasting interactions between DCs and Tregs were dependent on Nrp1 since the incubation of either DCs or Tregs with a Nrp1 blocking antibody reduced their interactions. This finding suggests that homotypic expression of Nrp1 by DCs and Tregs may contribute to this process. Further analyses demonstrated that Nrp1 plays a role in immune synapse formation (being present in pSMAC and cSMAC) as well as in antigen recognition and T-cell proliferation, since Nrp1 expression would make T cells more sensitive to antigen (23). Although the data described above suggests that Nrp1 is preferentially expressed by Tregs, Milpied et al. reported that human PBMC CD4+CD25+Foxp3+ T cells express extremely low levels of Nrp1, concluding that Nrp1 cannot be used to distinguish human Tregs from conventional CD4+ T cells (82).

Besides the chemoattractant property between DC-T cells and its role in the formation of the immunological synapse, Glinka and Prud’homme proposed a role for Nrp1 as a transforming growth factor-β (TGF-β) receptor (83). They designed an Nrp1-Fc molecule, which when added to T cells in culture could bind to LAP-TGFβ or active TGFβ. Importantly, CD4+Nrp1− T cells bind to LAP-TGFβ via Nrp1-Fc gaining suppressive capacity.

The *in vivo* importance of Nrp1 expression on CD4+ T cells was determined by Solomon et al. through analysis of Nrp-1^flox/flox^CD4^Cre^ conditional knockout mice where both CD4+ and CD8+ T cells lack Nrp1 (84). These mice have normal T-cell development, including normal levels of Foxp3 expression in CD4+CD25+ T cells compared with control littermates. Nrp-1^flox/flox^CD4^Cre^ mice developed a more severe EAE characterized by CD4+ T cells skewed toward a Th17 phenotype, given by an up-regulation in the expression of RORγτ and enhanced IL-17 secretion. CD4+CD25+ T cells from the Nrp-1^flox/flox^CD4^Cre^ mice display a lack of their suppressive function despite normal Foxp3 expression levels. The suppression displayed by CD4+Nrp1+ T cells seems to be dependent on TGF-β since the inclusion of a TGF-β blocking antibody into DC-CD4+ T-cell co-cultures, in the presence of CD4+Nrp1+ T cells, abrogates suppression (84). This result was not obtained when an IL-10 blocking antibody was evaluated.

In 2012, two independent research groups reported that Nrp1 could be a useful surface marker to differentiate between natural-Tregs (nTregs) and induced-Tregs (iTregs) (85, 86). Using several transgenic mouse strains, Yadav et al. demonstrated that iTregs express very low levels of Nrp1. Yadav’s group has developed an *in vivo* model (based on EAE) in which the offspring of myelin-binding protein (MBP)-TCR-Tg mice crossed with RAG-KO mice lack nTregs, but iTregs can be detected in the periphery. In addition to this, they also tested the low dose-antigen immunization model to induce iTregs. In their experiments, they could identify and isolate iTregs to compare differentially expressed genes by gene arrays analysis, with the nTregs counterpart as control. Results from these experiments demonstrated that Nrp1 is preferentially expressed by nTregs. Nrp1− T cells express Foxp3 and have suppressive function *in vitro* and *in vivo* (85).

Exploiting *in vivo* models of iTreg generation in mucosa (lung and intestine), Weiss et al. observed the same phenomena but additionally, they tested for possible factors controlling Nrp1 expression. In this attempt, the group identified TGF-β and IL-6 to induce and inhibit Nrp1 expression, respectively (86).

Conversely, Hansen et al. utilized an *in vivo* tumor model to show that the lack of Nrp1 on CD4+ T cells does not allow tumor growth, mainly by eliciting the effector function of intra-tumoral CD8+ T cells (87). The depletion of Foxp3+ T cells and disruption of VEGF production on tumor cells mimic the phenotype seen in CD4+ T-cell specific Nrp1-deficient mice. Taken together, these data indicate that tumor-derived VEGF is required to attract CD4+Foxp3+Nrp1+ T cells that can support tumor growth. In the same line, Delgoffe et al. utilized mice with a Foxp3-specific Nrp1 deletion, and found that Nrp1 potentiates Treg cell function and stability both *in vitro* and *in vivo* via interaction with Sema4A expressed in pDCs (67). The Nrp1-Sema4A binding would allow the interaction between Nrp1 and the cytoplasmic domain of PTEN, inhibiting Akt-mTOR signaling, and favoring Foxo3a nuclear localization, which promotes Foxp3-dependent Treg survival, stability, and function.

### Implications in clinical transplantation tolerance

Currently in the clinical transplantation field, a molecular marker to identify and track Tregs is lacking because Foxp3 (the hallmark for Tregs) is an intracellular protein, thus it is not a useful tool to identify and isolate viable Tregs. Furthermore, Foxp3 expression in human Tregs is not characteristic of these suppressor cells compared to murine Tregs (82). Yadav and Weiss’s recent reports propose Nrp1 as an attractive new surface marker to distinguish murine Tregs from effector CD4+ T cells, even more, to discriminate between those generated in the periphery (iTregs) from the ones differentiated in the thymus or nTregs (85, 86).

In murine studies, the contribution of Nrp1 biology in transplantation tolerance indicates that CD4+Nrp1+ T cells may play a suppressive role in allograft rejection. One important report shows that CD4+Nrp1+ T cells transferred into heart allograft-recipient mice extend the survival of the transplant, mainly by inhibiting the production of inflammatory cytokines, such as IFN-γ and IL-17, enriching for Tregs and inducing anergy on effector T cells (88). The suppressive capabilities of the CD4+Nrp1+ T cells were heightened by the co-administration of the immunosuppressant, rapamycin. Taken together, these results demonstrate the potential use of Nrp1+ T cells to abolish or diminish allograft effector responses, which can be complemented with current drugs to maximize the tolerogenic effect. In line with this report, Schliesser et al. generated CD4+CD25+Foxp3+ Treg cells from total mouse CD4+ T-cell *in vitro* through treatments with anti-CD4, TGF-β, and Retinoic acid (RA) or anti-CD4 plus rapamycin, and showed that the majority of Foxp3-expressing Tregs generated under anti-CD4, TGF-β, and RA condition express Nrp1, mainly due to the expansion of nTregs instead of *de novo* iTregs (89). They also showed that anti-CD4, TGF-β, and RA expanded Tregs exhibited the highest stability and suppressive capacities both *in vitro* and in a skin transplantation setting, highlighting the role of Nrp1 in Treg cells.

In humans, Nrp1 may be a useful indicator of the immunological status of patients. For example, Battaglia et al. analyzed the frequencies of CD4+Nrp1+ T cells in peripheral blood and lymph nodes of patients with benign diseases undergoing lymphadenectomy. Their results show that Foxp3 expression is detected in the CD25high fraction of CD4+Nrp1+ T cells from lymph nodes and not from blood, suggesting an anatomical influence in the presence of Nrp1-expressing CD4+ T cells (90). Importantly, CD4+Nrp1+CD25high T cells from lymph nodes co-expressed other Treg-like markers such as CD45RO and GITR, and displayed suppressive capability *in vitro*. Interestingly, the frequencies of CD4+Nrp1+CD25high T cells were reduced in the tumor-draining lymph nodes of cervical cancer patients after chemoradiation therapy, which was associated with a decrease in the tumor mass. Based on this data, one possibility is that the presence of human CD4+Nrp1+CD25high T in lymph nodes cooperates with the tolerogenic microenvironment of tumor burden areas (90).

**Figure 3:**
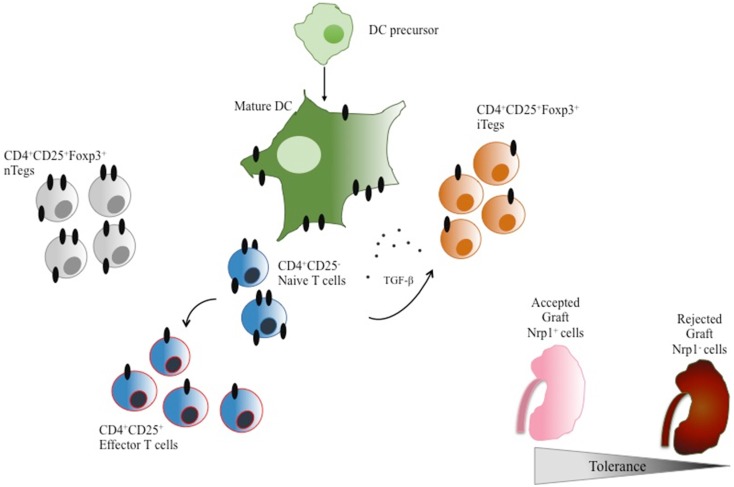
**Neuropilin-1 expression in DCs and CD4+ T cells**. DC precursors express very low levels of Nrp1. In contrast, mature DCs express high levels of Nrp1. Naïve CD4+CD25− T cells constitutively express Nrp1, which is downregulated upon T-cell activation. Interestingly, nTregs also constitutively express high levels of Nrp1 in contrast to iTregs, distinguishing these two cell populations when combined with Foxp3 expression. The presence of Nrp1+ T cells is associated with acceptance of tissue grafts while Nrp1− T cells are linked to tissue graft rejection.

Finally, a small study proposed the expression of Nrp1 on T cells as a putative predictor of allograft rejection, since the presence of Nrp1 on lymphocytes residing in kidney transplant biopsies decreased during acute rejection, as compared with biopsies from non-rejecting individuals (91), suggesting that the reduction of Tregs (Nrp1+ cells) in the graft may be linked with the development of the rejection process (see Figure 3 for an overview of Nrp1 expression on suppressive and effectors CD4+ T cells). This supports the possible use of Nrp1 as a Tregs marker in transplanted patients.

## Conclusion

Important advances have been made in the study of Nrp1 on immune cells. While most of the available information indicates a role for Nrp1 in the formation of the immunological synapse, Nrp1 may also play a key role as a Treg marker, permitting the discrimination between nTregs and periphery-induced-Tregs. More investigation is required to clearly demonstrate that Nrp1 will be a feasible predictor of tolerance in the transplantation field since murine and human data are not compatible. Hence, the question of whether Nrp1 is a new marker for transplantation tolerance remains to be answered.

## Author Contributions

Mauricio Campos-Mora wrote the manuscript and designed the figures, Rodrigo A. Morales wrote the manuscript and edited the figures, Tania Gajardo wrote the manuscript and designed the figures, Diego Catalán wrote the manuscript, and Karina Pino-Lagos wrote the manuscript and designed the figures.

## Conflict of Interest Statement

The authors declare that the research was conducted in the absence of any commercial or financial relationships that could be construed as a potential conflict of interest.

## References

[1] Parker MW Guo H-F Li X Linkugel AD Kooi CWV Function of members of the neuropilin family as essential pleiotropic cell surface receptors. Biochemistry (2012) 51(47):9437–46 10.1021/bi3012143 23116416 PMC3510667

[2] Gu C Limberg BJ Whitaker GB Perman B Leahy DJ Rosenbaum JS Characterization of neuropilin-1 structural features that confer binding to semaphorin 3A and vascular endothelial growth factor 165. J Biol Chem (2002) 277(20):18069–76 10.1074/jbc.M201681200 11886873

[3] Geretti E Shimizu A Klagsbrun M Neuropilin structure governs VEGF and semaphorin binding and regulates angiogenesis. Angiogenesis (2008) 11(1):31–9 10.1007/s10456-008-9097-1 18283547

[4] Roth L Nasarre C Dirrig-Grosch S Aunis D Cremel G Hubert P Transmembrane domain interactions control biological functions of neuropilin-1. Mol Biol Cell (2008) 19(2):646–54 10.1091/mbc.E07-06-0625 18045991 PMC2230599

[5] Cai H Reed RR Cloning and characterization of neuropilin-1-interacting protein: a PSD-95/Dlg/ZO-1 domain-containing protein that interacts with the cytoplasmic domain of neuropilin-1. J Neurosci (1999) 19(15):6519–27 10414980 10.1523/JNEUROSCI.19-15-06519.1999 PMC6782790

[6] Takagi S Tsuji T Amagai T Takamatsu T Fujisawa H Specific cell surface labels in the visual centers of *Xenopus laevis* tadpole identified using monoclonal antibodies. Dev Biol (1987) 122(1):90–100 3297854 10.1016/0012-1606(87)90335-6

[7] Takagi S Hirata T Agata K Mochii M Eguchi G Fujisawa H The A5 antigen, a candidate for the neuronal recognition molecule, has homologies to complement components and coagulation factors. Neuron (1991) 7(2):295–307 10.1016/0896-6273(91)90268-5 1908252

[8] Fujisawa H Discovery of semaphorin receptors, neuropilin and plexin, and their functions in neural development. J Neurobiol (2004) 59(1):24–33 10.1002/neu.10337 15007824

[9] Fujisawa H Takagi S Hirata T Growth-associated expression of a membrane protein, neuropilin, in Xenopus optic nerve fibers. Dev Neurosci (1995) 17(5–6):343–9 10.1159/000111304 8829923

[10] Kawakami A Kitsukawa T Takagi S Fujisawa H Developmentally regulated expression of a cell surface protein, neuropilin, in the mouse nervous system. J Neurobiol (1996) 29(1):1–17 10.1002/(SICI)1097-4695(199601)29:1<1::AID-NEU1>3.0.CO;2-F 8748368

[11] Kitsukawa T Shimono A Kawakami A Kondoh H Fujisawa H Overexpression of a membrane protein, neuropilin, in chimeric mice causes anomalies in the cardiovascular system, nervous system and limbs. Development (1995) 121(12):4309–18 8575331 10.1242/dev.121.12.4309

[12] Soker S Takashima S Miao HQ Neufeld G Klagsbrun M Neuropilin-1 is expressed by endothelial and tumor cells as an isoform-specific receptor for vascular endothelial growth factor. Cell (1998) 92(6):735–45 10.1016/S0092-8674(00)81402-6 9529250

[13] West DC Rees CG Duchesne L Patey SJ Terry CJ Turnbull JE Interactions of multiple heparin binding growth factors with neuropilin-1 and potentiation of the activity of fibroblast growth factor-2. J Biol Chem (2005) 280(14):13457–64 10.1074/jbc.M410924200 15695515

[14] Matsushita A Gotze T Korc M Hepatocyte growth factor-mediated cell invasion in pancreatic cancer cells is dependent on neuropilin-1. Cancer Res (2007) 67(21):10309–16 10.1158/0008-5472.CAN-07-3256 17974973

[15] Ball SG Bayley C Shuttleworth CA Kielty CM Neuropilin-1 regulates platelet-derived growth factor receptor signalling in mesenchymal stem cells. Biochem J (2010) 427(1):29–40 10.1042/BJ20091512 20102335 PMC3441150

[16] Pellet-Many C Frankel P Evans IM Herzog B Junemann-Ramirez M Zachary IC Neuropilin-1 mediates PDGF stimulation of vascular smooth muscle cell migration and signalling via p130Cas. Biochem J (2011) 435(3):609–18 10.1042/BJ20100580 21306301 PMC3086270

[17] Hsieh SH Ying NW Wu MH Chiang WF Hsu CL Wong TY Galectin-1, a novel ligand of neuropilin-1, activates VEGFR-2 signaling and modulates the migration of vascular endothelial cells. Oncogene (2008) 27(26):3746–53 10.1038/sj.onc.1211029 18223683

[18] Fukasawa M Matsushita A Korc M Neuropilin-1 interacts with integrin β1 and modulates pancreatic cancer cell growth, survival and invasion. Cancer Biol Ther (2007) 6:1184–91 10.4161/cbt.6.8.4363 17726369

[19] Robinson SD Reynolds LE Kostourou V Reynolds AR da Silva RG Tavora B Alphav beta3 integrin limits the contribution of neuropilin-1 to vascular endothelial growth factor-induced angiogenesis. J Biol Chem (2009) 284(49):33966–81 10.1074/jbc.M109.030700 19837659 PMC2797167

[20] Tordjman R Lepelletier Y Lemarchandel V Cambot M Gaulard P Hermine O A neuronal receptor, neuropilin-1, is essential for the initiation of the primary immune response. Nat Immunol (2002) 3(5):477–82 11953749 10.1038/ni789

[21] Corbel C Lemarchandel V Thomas-Vaslin V Pelus AS Agboton C Roméo PH Neuropilin 1 and CD25 co-regulation during early murine thymic differentiation. Dev Comp Immunol (2007) 31(11):1082–94 10.1016/j.dci.2007.01.009 17374393

[22] Bruder D Probst-Kepper M Westendorf AM Geffers R Beissert S Loser K Neuropilin-1: a surface marker of regulatory T cells. Eur J Immunol (2004) 34(3):623–30 10.1002/eji.200324799 14991591

[23] Sarris M Andersen KG Randow F Mayr L Betz AG Neuropilin-1 expression on regulatory T cells enhances their interactions with dendritic cells during antigen recognition. Immunity (2008) 28(3):402–13 10.1016/j.immuni.2008.01.012 18328743 PMC2726439

[24] Hirata T Takagi S Fujisawa H The membrane protein A5, a putative neuronal recognition molecule, promotes neurite outgrowth. Neurosci Res (1993) 17(2):159–69 10.1016/0168-0102(93)90092-5 8233120

[25] He Z Tessier-Lavigne M Neuropilin is a receptor for the axonal chemorepellent semaphorin III. Cell (1997) 90(4):739–51 10.1016/S0092-8674(00)80534-6 9288753

[26] Kolodkin AL Levengood DV Rowe EG Tai YT Giger RJ Ginty DD Neuropilin is a semaphorin III receptor. Cell (1997) 90(4):753–62 10.1016/S0092-8674(00)80535-8 9288754

[27] Kitsukawa T Shimizu M Sanbo M Hirata T Taniguchi M Bekku Y Neuropilin-semaphorin III/D-mediated chemorepulsive signals play a crucial role in peripheral nerve projection in mice. Neuron (1997) 19(5):995–1005 10.1016/S0896-6273(00)80392-X 9390514

[28] Kawasaki T Kitsukawa T Bekku Y Matsuda Y Sanbo M Yagi T A requirement for neuropilin-1 in embryonic vessel formation. Development (1999) 126(21):4895–902 10518505 10.1242/dev.126.21.4895

[29] Chen H Chedotal A He Z Goodman CS Tessier-Lavigne M Neuropilin-2, a novel member of the neuropilin family, is a high affinity receptor for the semaphorins Sema E and Sema IV but not Sema III. Neuron (1997) 19(3):547–59 10.1016/S0896-6273(00)80371-2 9331348

[30] Pellet-Many C Frankel P Jia H Zachary I Neuropilins: structure, function and role in disease. Biochem J (2008) 411(2):211–26 10.1042/BJ20071639 18363553

[31] Prud’homme GJ Glinka Y Neuropilins are multifunctional coreceptors involved in tumor initiation, growth, metastasis and immunity. Oncotarget (2012) 3(9):921–39 22948112 10.18632/oncotarget.626 PMC3660061

[32] Chen H He Z Bagri A Tessier-Lavigne M Semaphorin-neuropilin interactions underlying sympathetic axon responses to class III semaphorins. Neuron (1998) 21(6):1283–90 10.1016/S0896-6273(00)80648-0 9883722

[33] Herzog B Pellet-Many C Britton G Hartzoulakis B Zachary IC VEGF binding to NRP1 is essential for VEGF stimulation of endothelial cell migration, complex formation between NRP1 and VEGFR2, and signaling via FAK Tyr407 phosphorylation. Mol Biol Cell (2011) 22(15):2766–76 10.1091/mbc.E09-12-1061 21653826 PMC3145551

[34] Nakamura F Goshima Y Structural and functional relation of neuropilins. Adv Exp Med Biol (2002) 515:55–69 10.1007/978-1-4615-0119-0_5 12613543

[35] Gagnon ML Bielenberg DR Gechtman Z Miao HQ Takashima S Soker S Identification of a natural soluble neuropilin-1 that binds vascular endothelial growth factor: in vivo expression and antitumor activity. Proc Natl Acad Sci U S A (2000) 97(6):2573–8 10.1073/pnas.040337597 10688880 PMC15970

[36] Rossignol M Gagnon ML Klagsbrun M Genomic organization of human neuropilin-1 and neuropilin-2 genes: identification and distribution of splice variants and soluble isoforms. Genomics (2000) 70(2):211–22 10.1006/geno.2000.6381 11112349

[37] Cackowski FC Xu L Hu B Cheng SY Identification of two novel alternatively spliced Neuropilin-1 isoforms. Genomics (2004) 84(1):82–94 10.1016/j.ygeno.2004.02.001 15203206 PMC2868064

[38] Mamluk R Gechtman Z Kutcher ME Gasiunas N Gallagher J Klagsbrun M Neuropilin-1 binds vascular endothelial growth factor 165, placenta growth factor-2, and heparin via its b1b2 domain. J Biol Chem (2002) 277(27):24818–25 10.1074/jbc.M200730200 11986311

[39] Partanen TA Vuola P Jauhiainen S Lohi J Salminen P Pitkaranta A Neuropilin-2 and vascular endothelial growth factor receptor-3 are up-regulated in human vascular malformations. Angiogenesis (2013) 16(1):137–46 10.1007/s10456-012-9305-x 22961441

[40] Bates DO Harper SJ Regulation of vascular permeability by vascular endothelial growth factors. Vascul Pharmacol (2002) 39(4–5):225–37 10.1016/S1537-1891(03)00011-9 12747962

[41] Wild JR Staton CA Chapple K Corfe BM Neuropilins: expression and roles in the epithelium. Int J Exp Pathol (2012) 93(2):81–103 10.1111/j.1365-2613.2012.00810.x 22414290 PMC3385701

[42] Hasan NM Kendrick MA Druckenbrod NR Huelsmeyer MK Warner TF MacDonald MJ Genetic association of the neuropilin-1 gene with type 1 diabetes in children: neuropilin-1 expression in pancreatic islets. Diabetes Res Clin Pract (2010) 87(3):e29–32 10.1016/j.diabres.2009.12.016 20053475

[43] Roche J Drabkin H Brambilla E Neuropilin and its ligands in normal lung and cancer. Adv Exp Med Biol (2002) 515:103–14 10.1007/978-1-4615-0119-0_9 12613547

[44] Robert B Zhao X Abrahamson DR Coexpression of neuropilin-1, Flk1, and VEGF(164) in developing and mature mouse kidney glomeruli. Am J Physiol Renal Physiol (2000) 279(2):F275–82 10919846 10.1152/ajprenal.2000.279.2.F275

[45] Kumar I Staton CA Cross SS Reed MW Brown NJ Angiogenesis, vascular endothelial growth factor and its receptors in human surgical wounds. Br J Surg (2009) 96(12):1484–91 10.1002/bjs.6778 19918856

[46] Klagsbrun M Takashima S Mamluk R The role of neuropilin in vascular and tumor biology. Adv Exp Med Biol (2002) 515:33–48 10.1007/978-1-4615-0119-0_3 12613541

[47] Lacal PM Failla CM Pagani E Odorisio T Schietroma C Falcinelli S Human melanoma cells secrete and respond to placenta growth factor and vascular endothelial growth factor. J Invest Dermatol (2000) 115(6):1000–7 10.1046/j.1523-1747.2000.00199.x 11121133

[48] Ding H Wu X Roncari L Lau N Shannon P Nagy A Expression and regulation of neuropilin-1 in human astrocytomas. Int J Cancer (2000) 88(4):584–92 10.1002/1097-0215(20001115)88:4<584::AID-IJC11>3.0.CO;2-T 11058875

[49] Latil A Bieche I Pesche S Valeri A Fournier G Cussenot O VEGF overexpression in clinically localized prostate tumors and neuropilin-1 overexpression in metastatic forms. Int J Cancer (2000) 89(2):167–71 10.1002/(SICI)1097-0215(20000320)89:2<167::AID-IJC11>3.0.CO;2-9 10754495

[50] Kikutani H Kumanogoh A Semaphorins in interactions between T cells and antigen-presenting cells. Nat Rev Immunol (2003) 3(2):159–67 10.1038/nri1003 12563299

[51] Potiron V Nasarre P Roche J Healy C Boumsell L Semaphorin signaling in the immune system. Adv Exp Med Biol (2007) 600:132–44 17607952 10.1007/978-0-387-70956-7_11

[52] Tamagnone L Artigiani S Chen H He Z Ming GI Song H Plexins are a large family of receptors for transmembrane, secreted, and GPI-anchored semaphorins in vertebrates. Cell (1999) 99(1):71–80 10.1016/S0092-8674(00)80063-X 10520995

[53] Kumanogoh A Kikutani H Immune semaphorins: a new area of semaphorin research. J Cell Sci (2003) 116(Pt 17):3463–70 10.1242/jcs.00674 12893810

[54] Takamatsu H Kumanogoh A Diverse roles for semaphorin-plexin signaling in the immune system. Trends Immunol (2012) 33(3):127–35 10.1016/j.it.2012.01.008 22325954

[55] Takegahara N Kumanogoh A Kikutani H Semaphorins: a new class of immunoregulatory molecules. Philos Trans R Soc Lond B Biol Sci (2005) 360(1461):1673–80 10.1098/rstb.2005.1696 16147531 PMC1569539

[56] O’Connor BP Eun SY Ye Z Zozulya AL Lich JD Moore CB Semaphorin 6D regulates the late phase of CD4+ T cell primary immune responses. Proc Natl Acad Sci U S A (2008) 105(35):13015–20 10.1073/pnas.0803386105 18728195 PMC2529027

[57] Zhao XY Chen L Xu Q Li YH Expression of semaphorin 6D in gastric carcinoma and its significance. World J Gastroenterol (2006) 12(45):7388–90 17143962 10.3748/wjg.v12.i45.7388 PMC4087504

[58] Delaire S Elhabazi A Bensussan A Boumsell L CD100 is a leukocyte semaphorin. Cell Mol Life Sci (1998) 54(11):1265–76 10.1007/s000180050252 9849618 PMC11147245

[59] Shi W Kumanogoh A Watanabe C Uchida J Wang X Yasui T The class IV semaphorin CD100 plays nonredundant roles in the immune system: defective B and T cell activation in CD100-deficient mice. Immunity (2000) 13(5):633–42 10.1016/S1074-7613(00)00063-7 11114376

[60] Kumanogoh A Suzuki K Ch’ng E Watanabe C Marukawa S Takegahara N Requirement for the lymphocyte semaphorin, CD100, in the induction of antigen-specific T cells and the maturation of dendritic cells. J Immunol (2002) 169(3):1175–81 12133937 10.4049/jimmunol.169.3.1175

[61] Adachi T Flaswinkel H Yakura H Reth M Tsubata T Cutting edge: the B cell surface protein CD72 recruits the tyrosine phosphatase SHP-1 upon tyrosine phosphorylation. J Immunol (1998) 160(10):4662–5 9590210

[62] Wu Y Nadler MJ Brennan LA Gish GD Timms JF Fusaki N The B-cell transmembrane protein CD72 binds to and is an in vivo substrate of the protein tyrosine phosphatase SHP-1. Curr Biol (1998) 8(18):1009–17 10.1016/S0960-9822(07)00421-6 9740800

[63] Kumanogoh A Watanabe C Lee I Wang X Shi W Araki H Identification of CD72 as a lymphocyte receptor for the class IV semaphorin CD100: a novel mechanism for regulating B cell signaling. Immunity (2000) 13(5):621–31 10.1016/S1074-7613(00)00062-5 11114375

[64] Kumanogoh A Marukawa S Suzuki K Takegahara N Watanabe C Ch’ng E Class IV semaphorin Sema4A enhances T-cell activation and interacts with Tim-2. Nature (2002) 419(6907):629–33 10.1038/nature01037 12374982

[65] Kumanogoh A Shikina T Suzuki K Uematsu S Yukawa K Kashiwamura S Nonredundant roles of Sema4A in the immune system: defective T cell priming and Th1/Th2 regulation in Sema4A-deficient mice. Immunity (2005) 22(3):305–16 10.1016/j.immuni.2005.01.014 15780988

[66] Nakatsuji Y Okuno T Moriya M Sugimoto T Kinoshita M Takamatsu H Elevation of Sema4A implicates Th cell skewing and the efficacy of IFN-beta therapy in multiple sclerosis. J Immunol (2012) 188(10):4858–65 10.4049/jimmunol.1102023 22491253

[67] Delgoffe GM Woo SR Turnis ME Gravano DM Guy C Overacre AE Stability and function of regulatory T cells is maintained by a neuropilin-1-semaphorin-4a axis. Nature (2013) 501(7466):252–6 10.1038/nature12428 23913274 PMC3867145

[68] Nakagawa Y Takamatsu H Okuno T Kang S Nojima S Kimura T Identification of semaphorin 4B as a negative regulator of basophil-mediated immune responses. J Immunol (2011) 186(5):2881–8 10.4049/jimmunol.1003485 21270411

[69] Choi YI Duke-Cohan JS Ahmed WB Handley MA Mann F Epstein JA PlexinD1 glycoprotein controls migration of positively selected thymocytes into the medulla. Immunity (2008) 29(6):888–98 10.1016/j.immuni.2008.10.008 19027330 PMC2615553

[70] Lepelletier Y Moura IC Hadj-Slimane R Renand A Fiorentino S Baude C Immunosuppressive role of semaphorin-3A on T cell proliferation is mediated by inhibition of actin cytoskeleton reorganization. Eur J Immunol (2006) 36(7):1782–93 10.1002/eji.200535601 16791896

[71] Catalano A Caprari P Moretti S Faronato M Tamagnone L Procopio A Semaphorin-3A is expressed by tumor cells and alters T-cell signal transduction and function. Blood (2006) 107(8):3321–9 10.1182/blood-2005-06-2445 16380453

[72] Takahashi T Fournier A Nakamura F Wang LH Murakami Y Kalb RG Plexin-neuropilin-1 complexes form functional semaphorin-3A receptors. Cell (1999) 99(1):59–69 10.1016/S0092-8674(00)80062-8 10520994

[73] Yamamoto M Suzuki K Okuno T Ogata T Takegahara N Takamatsu H Plexin-A4 negatively regulates T lymphocyte responses. Int Immunol (2008) 20(3):413–20 10.1093/intimm/dxn006 18209113

[74] Catalano A The neuroimmune semaphorin-3A reduces inflammation and progression of experimental autoimmune arthritis. J Immunol (2010) 185(10):6373–83 10.4049/jimmunol.0903527 20937848

[75] Vadasz Z Haj T Halasz K Rosner I Slobodin G Attias D Semaphorin 3A is a marker for disease activity and a potential immunoregulator in systemic lupus erythematosus. Arthritis Res Ther (2012) 14(3):R146 10.1186/ar3881 22697500 PMC3446531

[76] Eixarch H Gutierrez-Franco A Montalban X Espejo C Semaphorins 3A and 7A: potential immune and neuroregenerative targets in multiple sclerosis. Trends Mol Med (2013) 19(3):157–64 10.1016/j.molmed.2013.01.003 23419749

[77] Lepelletier Y Smaniotto S Hadj-Slimane R Villa-Verde DM Nogueira AC Dardenne M Control of human thymocyte migration by Neuropilin-1/Semaphorin-3A-mediated interactions. Proc Natl Acad Sci U S A (2007) 104(13):5545–50 10.1073/pnas.0700705104 17369353 PMC1838472

[78] Delaire S Billard C Tordjman R Chedotal A Elhabazi A Bensussan A Biological activity of soluble CD100. II. Soluble CD100, similarly to H-SemaIII, inhibits immune cell migration. J Immunol (2001) 166(7):4348–54 11254688 10.4049/jimmunol.166.7.4348

[79] Ji JD Park-Min KH Ivashkiv LB Expression and function of semaphorin 3A and its receptors in human monocyte-derived macrophages. Hum Immunol (2009) 70(4):211–7 10.1016/j.humimm.2009.01.026 19480842 PMC4811352

[80] Takamatsu H Takegahara N Nakagawa Y Tomura M Taniguchi M Friedel RH Semaphorins guide the entry of dendritic cells into the lymphatics by activating myosin II. Nat Immunol (2010) 11(7):594–600 10.1038/ni.1885 20512151 PMC3045806

[81] Wen H Lei Y Eun SY Ting JP Plexin-A4-semaphorin 3A signaling is required for Toll-like receptor- and sepsis-induced cytokine storm. J Exp Med (2010) 207(13):2943–57 10.1084/jem.20101138 21098092 PMC3005237

[82] Milpied P Renand A Bruneau J Mendes-da-Cruz DA Jacquelin S Asnafi V Neuropilin-1 is not a marker of human Foxp3+ Treg. Eur J Immunol (2009) 39(6):1466–71 10.1002/eji.200839040 19499532

[83] Glinka Y Prud’homme GJ Neuropilin-1 is a receptor for transforming growth factor beta-1, activates its latent form, and promotes regulatory T cell activity. J Leukoc Biol (2008) 84(1):302–10 10.1189/jlb.0208090 18436584 PMC2504713

[84] Solomon BD Mueller C Chae WJ Alabanza LM Bynoe MS Neuropilin-1 attenuates autoreactivity in experimental autoimmune encephalomyelitis. Proc Natl Acad Sci U S A (2011) 108(5):2040–5 10.1073/pnas.1008721108 21245328 PMC3033275

[85] Yadav M Louvet C Davini D Gardner JM Martinez-Llordella M Bailey-Bucktrout S Neuropilin-1 distinguishes natural and inducible regulatory T cells among regulatory T cell subsets in vivo. J Exp Med (2012) 209(10):1713–22; s1711–19. 10.1084/jem.20120822 22966003 PMC3457729

[86] Weiss JM Bilate AM Gobert M Ding Y Curotto de Lafaille MA Parkhurst CN Neuropilin 1 is expressed on thymus-derived natural regulatory T cells, but not mucosa-generated induced Foxp3+ T reg cells. J Exp Med (2012) 209(10):1723–42 s1721, 10.1084/jem.20120914 22966001 PMC3457733

[87] Hansen W Hutzler M Abel S Alter C Stockmann C Kliche S Neuropilin 1 deficiency on CD4+Foxp3+ regulatory T cells impairs mouse melanoma growth. J Exp Med (2012) 209(11):2001–16 10.1084/jem.20111497 23045606 PMC3478934

[88] Yuan Q Hong S Shi B Kers J Li Z Pei X CD4(+)CD25(-)Nrp1(+) T cells synergize with rapamycin to prevent murine cardiac allorejection in immunocompetent recipients. PLoS One (2013) 8(4):e61151 10.1371/journal.pone.0061151 23577203 PMC3618334

[89] Schliesser U Chopra M Beilhack A Appelt C Vogel S Schumann J Generation of highly effective and stable murine alloreactive Treg cells by combined anti-CD4 mAb, TGF-β, and RA treatment. Eur J Immunol (2013). 10.1002/eji.201243292 23946112

[90] Battaglia A Buzzonetti A Monego G Peri L Ferrandina G Fanfani F Neuropilin-1 expression identifies a subset of regulatory T cells in human lymph nodes that is modulated by preoperative chemoradiation therapy in cervical cancer. Immunology (2008) 123(1):129–38 10.1111/j.1365-2567.2007.02737.x 18028372 PMC2433274

[91] Zhou H Zhang L Tong L Cai M Guo H Yang C Expression of neuropilin-1 in kidney graft biopsies: what is the significance? Transplant Proc (2007) 39(1):81–3 10.1016/j.transproceed.2006.10.221 17275479

